# Realization of the rights of persons with disabilities in Rwanda

**DOI:** 10.1371/journal.pone.0196347

**Published:** 2018-05-10

**Authors:** Janet Njelesani, Jenna Siegel, Emily Ullrich

**Affiliations:** Department of Occupational Therapy, New York University, New York, New York, United States of America; Universita degli Studi di Perugia, ITALY

## Abstract

This scoping study assessed the realization of the rights for persons with disabilities in Rwanda since the signing of the United Nations Convention on the Rights of Persons with Disabilities (UN CRPD) in 2008. Underpinned by the five-stage framework of Arksey and O’Malley, the scoping study examined peer-reviewed literature published between 2008 and 2017. Nine electronic databases were searched using keywords specific to disability in Rwanda. Data were charted by three reviewers according to pre-determined and emergent categories. Descriptive statistics were used to describe the data sources. A total of 60 scholarly articles met the inclusion criteria. Within the research, studies pertaining to the UN CRPD Articles of health, awareness raising, accessibility, and children with disabilities were the most published. The literature identified a movement towards the realization of the rights for persons with disabilities in Rwanda since the country signed the UN CRPD. Despite efforts to meet these rights, discrimination against persons with disabilities still exists and greater investment in the disability sector is needed, particularly for justice, social protection, and mental health services. Given the state of the evidence, concerning research gaps also exist in regards to deinstitutionalization and protection issues (i.e., violence and abuse). This consolidation of evidence may help to inform the decision-making priorities for government and civil society organizations in policy and programming and also direct future research.

## Introduction

The signing of the United Nations Convention on the Rights of Persons with Disabilities (UN CRPD) on May 3, 2008 is one of the most important disability actions taken in Rwanda to date. The signing of the UN CRPD marked national recognition of the rights for persons with disabilities (PWDs) and put into effect in Rwanda the first all-encompassing human rights treaty of the century. The signing of the UN CRPD is also viewed as a landmark in the history of the disability rights movement in Rwanda, as it represents the adoption of a social, rather than a medical, model of disability.

The UN CRPD was adopted by the United Nation General Assembly in 2006 as a human rights treaty, opened to signatories in 2007, and came into force in 2008. While the UN CRPD does not create any new rights or entitlements, it expresses existing rights in a mode that addresses the needs and situations of persons with disabilities. The UN CRPD details these specific rights of PWDs and also identifies areas where violations of these rights have occurred, along with ways in which society can adapt to support the realization of rights for PWDs [1]. Guiding principles are respect for inherent dignity, individual autonomy including the freedom to make one’s own choices, non-discrimination, full and effective participation and inclusion in society, equality of opportunity, and accessibility. The Convention is the culmination of the United Nations many years of work to increase worldwide awareness of individuals living with disabilities and place emphasis on changing maladaptive treatment approaches and negative attitudes. The ratifying states acknowledge that the principles of the UN CRPD should be transposed into their national legislations.

The UN CRPD has 50 Articles (Table 1). Articles 1–4 describe the purpose, definition, principles, and obligations of States Parties. Articles 5–9 detail principles on non-discrimination, accessibility, equality between men and women, and respecting the rights of children with disabilities. Articles 10–30 guarantee the rights of PWDs. Articles 24–28 entitle basic rights such as education, health, and employment. Articles 31–33 guide country implementation processes. Articles 34–50 address the process of ratification, government reporting, and cooperation between States Parties and the Committee on the Rights of Persons with Disabilities [2].

**Table 1:** Articles of the UN CRPD.

1. Purpose 2. Definitions 3. General Principles 4. General Obligations 5. Equality and Non-discrimination 6. Women with disabilities 7. Children with disabilities 8. Awareness raising 9. Accessibility 10. Right to life 11. Situations at risk and humanitarian emergency 12. Equal recognition before the law 13. Access to justice 14. Liberty and security of the person 15. Freedom from torture or cruel, inhuman or degrading treatment or punishment 16. Freedom from exploitation, violence and abuse 17. Protecting the integrity of the person 18. Liberty of movement and Nationality 19. Living independently and being included in the community 20. Personal mobility 21. Freedom of expression and opinion, and access to information 22. Respect for privacy 23. Respect for home and family 24. Education 25. Health 26. Habilitation and rehabilitation 27. Work and employment 28. Adequate standard of living and social protection 29. Participation in political and public life 30. Participation in cultural life, recreation, leisure and sport 31. Statistics and data collection 32. International cooperation 33. National implementation and monitoring 34. Committee on the rights of PWD 35. Reports by States Parties 36. Consideration of reports 37. Cooperation between States Parties and the Committee 38. Relationship of the Committee with other bodies 39. Report of the Committee 40. Conference of States Parties 41. Depository 42. Signature 43. Consent to be bound 44. Regional integration organization 45. Entry into force 46. Reservations 47. Amendments 48. Denunciation 49. Accessible format 50. Authentic texts

The ratification of the UN CRPD in Rwanda was a major step towards changing societal attitudes and approaches to PWDs. For many countries, ratification acts as a catalyst that spurs governmental development of inclusive policy, legislation, and planning. The disability community of Rwanda are hopeful that as a result of ratification of the UN CRPD there will be an even greater political will to change the status of PWDs and further advances in disability rights.

Prior to the signing of the UN CRPD, the evolution of the disability movement in Rwanda was characterized by faith based institutional care in the 1960s, the founding of disability associations including the Association Génèrale des Handicapés du Rwanda (AGHR) in the late 1970s, and then the formation of Disabled Peoples’ Organisations (DPOs) in the early 2000’s [3]. The 1994 Rwandan genocide is also significant to the history of disability in the country since it not only contributed to a greater prevalence of disability [4], but also shaped the disability movement. In its wake, there was an emergence of civil society groups aiming to rebuild and provide rehabilitation.

In the most recent National Population Census, the population of Rwanda is estimated to be 10.5 million. Approximately 446,000 Rwandans identify as PWDs, comprising 4.4 per cent of the population [5]. There is a small difference between genders, with a prevalence rate of 5.2 per cent for males (aged five and above) and 4.8 per cent for females. The World Health Organization estimates that 15 per cent of the world’s population constitutes persons living with disability [6], therefore these figures may be an underestimation. Rwandan law defines a disability as the state of a person who has lost the capacities that are essential to life or who has deficiencies compared to other persons and as a result does not enjoy equal chances and opportunities [7]. Under the terms of this law, PWDs are classified into the following categories: physically disabled, sight-impaired, deaf-and-dumb, mentally disabled, and persons not specified in the above. In Rwanda, poverty, disease, accidents, lack of medical care, and congenital causes account for the majority of disability [5]. The 1994 genocide also contributed to an increase in impairments not only as a direct result of the violence, but also because of the breakdown of health, vaccination, and rehabilitation services, as well as ongoing mental health conditions [4].

The disability movement efforts in Rwanda are guided by an equity-driven government agenda and supported by progressive policy efforts and legal governmental framework. Specific key initiatives include the National Policy on Disability in 2003, Law N° 01/2007 Relating to Protection of People with Disabilities in General, Law N° 02/2007 relating to the protection of disabled former war combatants, and Law N° 54/2011 which provides specific protections to children with disabilities. The National Council of Persons with Disabilities (NCPD) is another key initiative. Its mission is to be a forum for advocacy and social mobilization for issues affecting persons with disabilities in order to build PWD’s capacity and ensure their participation in the national development [3]. Many of these achievements have been attained in collaboration between the Government of Rwanda and civil society organizations, such as the National Union of Disabilities’ Organisations of Rwanda, UNICEF, and Handicap International.

## Methods

According to Arksey and O’Malley [8] researchers undertake scoping studies for various reasons including to examine the extent and nature of research activity; to identify whether a systematic review is necessary; to summarize and disseminate research findings; and to identify potential research gaps. A scoping study was chosen to guide this work in order to summarize literature in fields containing a paucity of rigorous evidence (i.e., disability rights in Rwanda), by incorporating literature that encompasses a broad range of study designs with the aim of drawing conclusions regarding the realization of rights of PWDs in Rwanda since the signing of the UN CRPD in 2008.

The approach for this scoping study is underpinned by the framework outlined by Arksey and O’Malley [8], which adopts a rigorous process of transparency, enabling replication of the search strategy and increasing the reliability of the study findings. This scoping study was conducted in five stages: identifying the research question, identifying relevant studies, study selection, charting the data, and collating, summarizing, and reporting the results.

### Stage 1: Identifying the research question

Since the signing of the UN CRPD in Rwanda, no comprehensive review of the realization of rights for PWDs in Rwanda has been conducted to understand the overall changes that have occurred as a result and note any gaps that may still remain. Therefore, the research question was: ‘What is known from the existing literature about the realization of the rights of PWDs since Rwanda signed the UN CRPD in 2008?’ This research question enabled the assessment of the degree to which research has kept pace with the progress being made in Rwanda since efforts to support the rights of PWDs were declared.

### Stage 2: Identifying relevant studies

Nine electronic databases, including SCOPUS, EMBASE, JSTOR, PsychINFO, ProQuest Central, CINAHL Plus, MEDLINE via PubMed, MEDLINE via Ovid, and the PAIS index were searched from January to April 2017. Arksey and O’Malley [8] suggest that a wide range of key words should be adopted to glean a broad coverage of available literature. The key constructs in the research question were used to develop a search string, which included an extensive collection of search terms. This study used both standardized subject (e.g., Medical Subject Headings (MeSH terms)) and free-text terms. To economize the search string, truncation (adding * to a word stem) was used in combination with free-text words. BOOLEAN searches were used to string specific keywords together for the database searches. The relevant MeSH terms, included: disabled children, disabled persons, Rwanda, civil rights, rehabilitation, health services accessibility, architectural accessibility. The following combination of free-text terms were used: Rwanda, disab*, impair*, handicap*, rehab*, access*, rights, persons living with disab*, person* with disab*, people with disab*, disab* person. A university librarian was also consulted who was a subject specialist in the field of rehabilitation. Their input was useful in the refinement of key search terms and identifying databases most likely to produce the results sought.

Each author performed an initial search of the databases and then later reviewed the work of the others to cross-reference for accuracy. As familiarity with the literature increased, keywords and databases used were adapted post-hoc to generate more relevant results.

### Stage 3: Study selection

Eligibility criteria for this scoping study was applied to condense the results to include exclusively persons with disabilities in Rwanda. Each database search filtered the publications using the following inclusion criteria:

1. Published during the years 2008 to 2017. The year 2008 was chosen because that is when Rwanda signed the UN CRPD.
2. Written in the English language.
3. Peer reviewed journal article.
4. Publication is about disability or contains information on at least one of the disability categories outlined by the Rwandan census.

**Fig 1:**
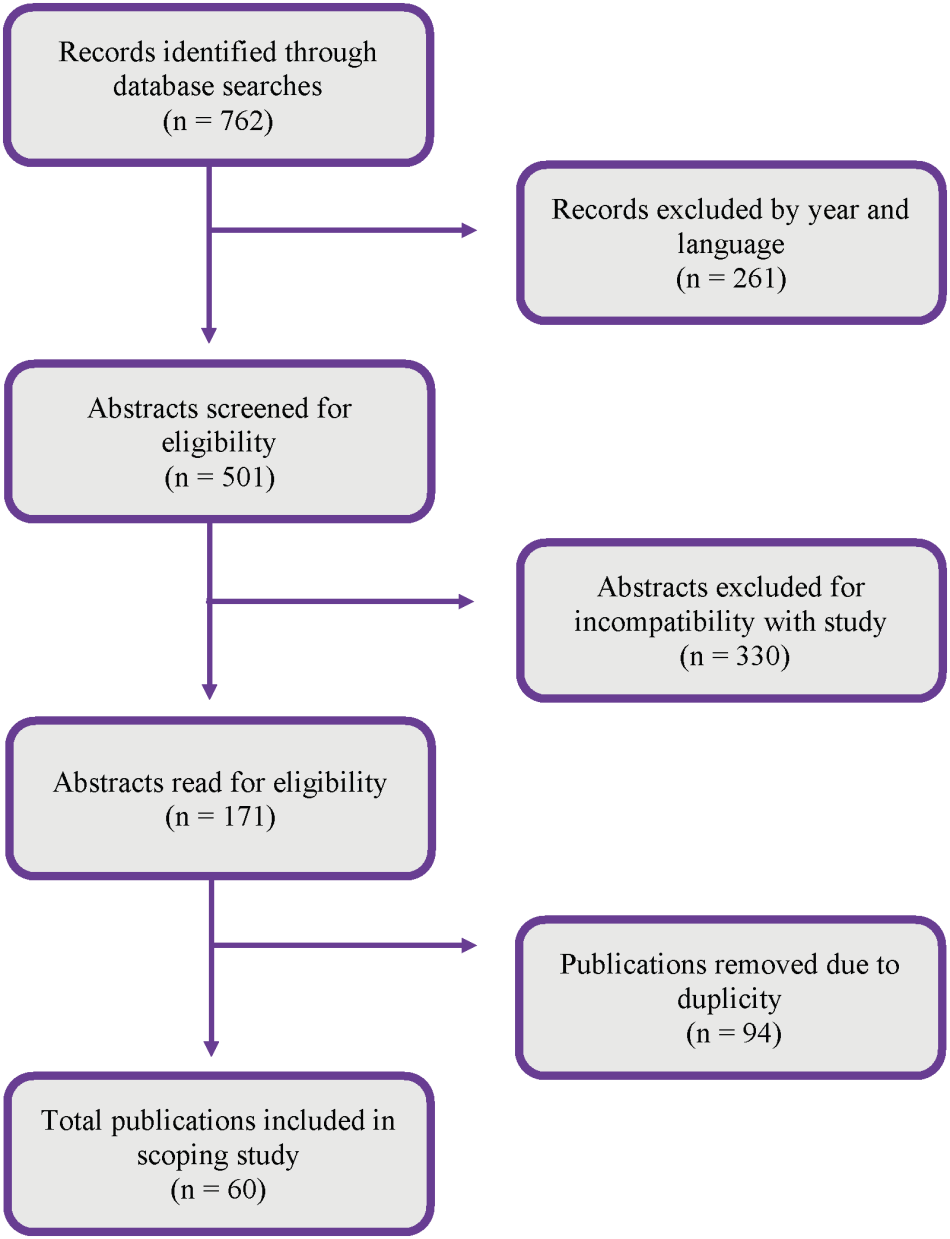
Study selection process.

Reference to the rights of PWDs was not included as part of the inclusion criteria because that was found to limit the search too narrowly when initially attempted. Prior to applying inclusion criteria, a total of 762 publications were generated in searches of the electronic databases. After setting the parameters for inclusion on the electronic databases, 501 publications remained. The individual abstracts of the remaining 501 publications were reviewed by two of the authors to determine their applicability to the research question. Each researcher initially reviewed the publications from half of the databases. If it was unclear if a publication should be included based solely on the abstract, the full-text publication was read to determine relevance. After the abstracts were read, 155 publications remained eligible for inclusion. The remaining 155 publications were cross-referenced by two of the authors for quality assurance and goodness of fit to the study. Doubts and disagreements were settled by discussion with the first author. Once abstracts were approved by the second and third author, copies of the full-text publications were downloaded for final approval by the first author and then uploaded to an online bibliographic citation management system (EndNote X8) for storage. This cross-referencing decreased the total amount of eligible studies by 1, leaving 154 publications. Duplicate publications were removed during stage 4 of the process, leaving a total of 60 publications. These 60 publications are included in this study. The study selection process is detailed in Fig 1 to illustrate how the final 60 publications were obtained.

### Stage 4: Charting the data

Each search was tracked using a chart that indicated which database was used, the specific keywords entered, the number of total hits generated, the number publications remaining after inclusion/exclusion criteria were applied, and the number of viable publications after abstracts were reviewed. An additional section was added post-hoc for the other authors to compare these values. A final column indicated the agreed upon number of publications that were applicable to this study.

Data extracted from the articles was compiled in a chart that detailed the publication’s title, author, year of publication, study population/type of disability, and associated right(s) of PWDs referenced. The associated right(s) for each publication was determined by the initial reviewer and later discussed between the second and third authors for confirmation. If it was unclear which associated right(s) fit for a publication, discussion with the first author and referencing the UN CRPD Articles determined which right(s) to include in the chart.

### Stage 5: Collating, summarizing, and reporting the results

The final stage of this scoping study was collating and finding patterns in the data from the information detailed in the charts using descriptive statistics to describe these data sources and generate a comprehensive understanding of the realization of rights for PWDs in Rwanda included in the literature over the last nine years. Using the UN CRPD as a guiding framework, data were categorized according to each Article of the UN CRPD and authors determined which right(s) were associated with the published literature.

The term positionality describes how people are defined, “not in terms of fixed identities, but by their location within shifting networks of relationships, which can be analyzed and changed” [9]. Based on this definition, positionality is always considered in relation to the place researchers occupy with respect to the world and is an important concept to explicitly articulate in research. The authors held multiple positionalities in relation to the topic of inquiry. We identify as female, non-African, English-speaking, nondisabled, occupational therapists living outside of Rwanda. The first author’s concurrent disability and rehabilitation research in Rwanda and time spent in country working with persons with disabilities and disabled persons organizations influenced the entire research process, including the interest in the topic, choice of research question, and decisions about what to include and what to omit.

## Results

From the initial 762 publications identified through database searches, 60 publications were determined to be eligible for inclusion in this study. The study population described in the 60 peer reviewed journal articles that met the inclusion criteria included 28 studies specific to children. The remainder pertained to adults and disability across all ages. The years of publication spread across the 9 years, with the most articles being published in 2016 (n = 10). A broad range of evidence exists in the articles. Articles represented differing research paradigms (e.g., positivist, interpretive), research designs (e.g., qualitative, mixed method, quantitative), and methods (e.g., survey, interview).

**Fig 2:**
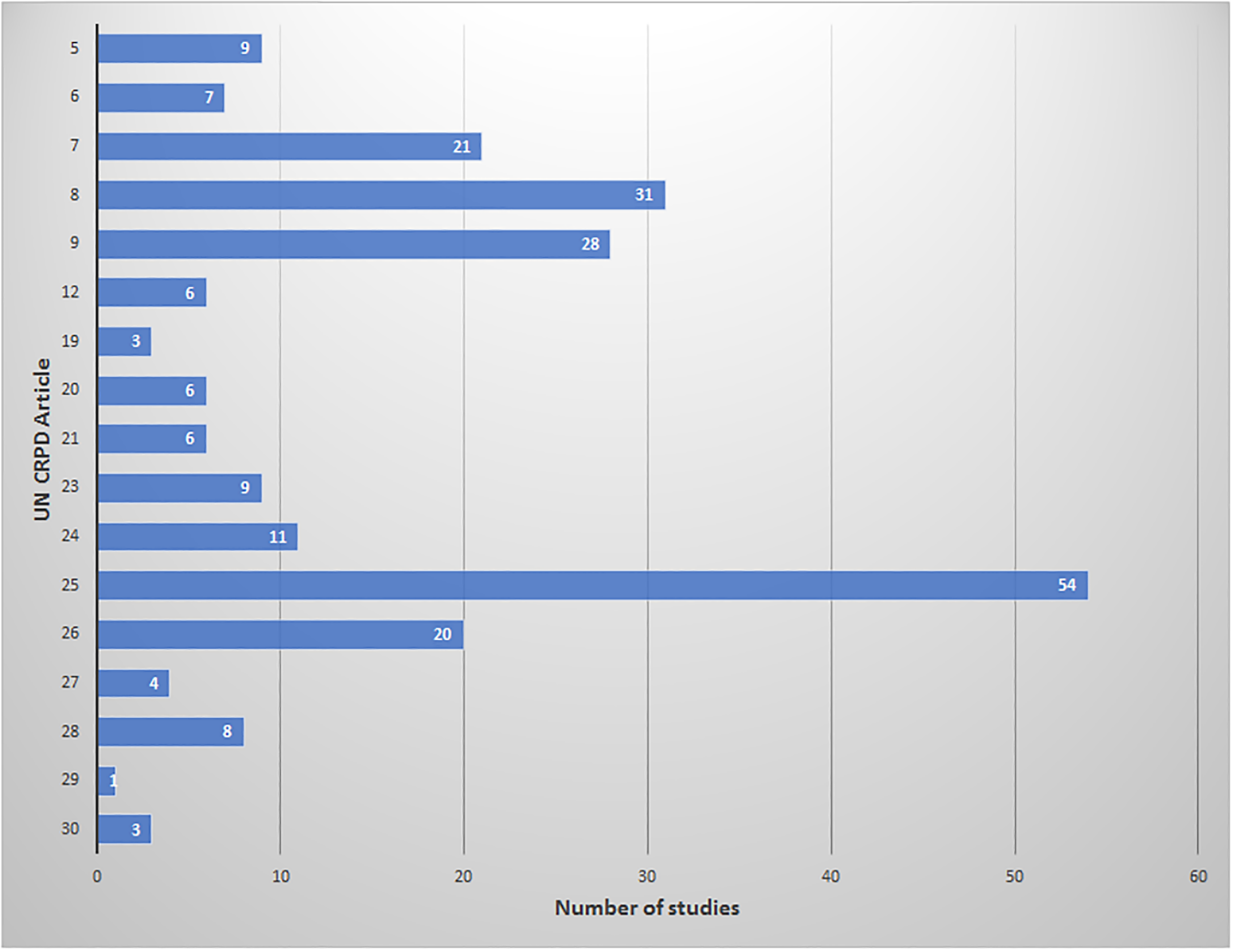
Number of studies published per UN CRPD Article.

Within the research, studies pertaining to Article 25 (Health), were the most published of any UN CRPD Article (n = 54) (Fig 2). Other UN CRPD Articles that were referenced in the literature most frequently included Article 7 (Children with Disabilities) (n = 21), Article 8 (Awareness-Raising) (n = 31), and Article 9 (Accessibility) (n = 28). Of the UN CRPD Articles, there were 10 (Articles 10, 11, 13, 14, 15, 16, 17, 18, 22, 31) that were not once mentioned within any of the publications reviewed. These Articles are representative of issues of justice (Articles 10, 13, 14, 17, 18, 22), violence and abuse (Articles 14, 15, 16), humanitarian response (Article 11), and disability data collection (Article 31), revealing under-studied areas of disability in Rwanda.

### Article 25 (Health)

In support of Article 25 (Health), Rwanda’s universal health insurance, Mutuelle de Santé, specifically aims to relieve some of the financial burdens of accessing healthcare. Despite this, the poorest parts of the population utilize health care less frequently than those who are financially stable. The introduction of higher premiums in 2010 made it challenging for people to pay for enrollment in the Mutuelle de Santé [10]. Additionally, PWDs still face obstacles because of a lack of healthcare funding and resources [11]. Specifically, there are not an adequate number of radiography machines for individuals with varying medical conditions, including but not limited to soft tissue or spinal cord injuries [12]. Moreover, due to the emerging economy in Rwanda, the costs of devices such as cochlear implants are challenging for most [13]. While Rwanda has achieved one of the highest coverage rates for antiretroviral HIV treatments in Africa [14], many infected PWDs still do not feel comfortable getting tested and many healthcare workers still feel uncomfortable offering the test [15]. The Rwandan Ministry of Health has created provisions to eliminate needless blindness by providing better eye care. This has included improvements in measurement and evaluation techniques, infrastructure, control of disease, and delivery of services. Nonetheless, it was determined that a better supply chain for obtaining eyewear and other assistive devices is still needed [16].

Many publications identified violations of rights of PWDs in Rwanda specific to the mental health sector. One of the underlying issues is that only 1 per cent of the national health budget is allocated to mental health needs [17]. Rugema, Krantz, Mogren, Ntaganira, and Persson [10] found that mental health services were underfinanced and described a situation in which “the total number of mental health nurses were 293 in 2012 and only two mental hospitals specialized in mental health care in Rwanda.” Additionally, it was found that as of 2014, no health centers within Rwanda had a quality assurance team or system to track mental health patient data [18]. However, enhancements to pre-existing programs such as the Rural Health Scale-Up National Strategic Plan have been initiated to address the need for mental health care in the country [19].

### Article 7 (Children with disabilities)

Abashize hamwe ntakibananira (nothing can defeat combined hands) was a central theme of the evidence of the realization of rights for children with disabilities. Resourceful communities and collective/communal support were cited abundantly in the literature [20,21], as children with disabilities were more visible, active members of their families [22].

Much of the literature that supported Article 7 interwove with Article 24 (Education). Under both Articles, the Ministry of Education’s universal primary education initiative to include children with disabilities in classrooms nationwide is key [23]. In support of this work, a publication by Byaruhanga and Kuteesa [24] stated that the National Council for Persons with Disabilities (NCPD) implemented a special needs policy in collaboration with the Ministry of Education. Additionally, Talley and Brintell [25] found that the Ministry of Education outlined standards to improve school setting conditions and Karangwa, Miles, and Lewis [22] identified governmental actions taken in the educational system to protect and assist children with disabilities at school. This strategic plan included “increasing public awareness about special education needs and disability, facilitating barrier-free learning environments, and supporting appropriate teacher development programmes.” Karangwa, Miles, and Lewis also found that teachers were aware of the benefits of including children with disabilities in schools. Despite these efforts made towards adherence with Articles 7 and 24, teachers remained fearful of teaching classes that included children with disabilities. For instance, children with epilepsy were often sent home because teachers were afraid that they would spread their condition to other children in the class [26]. Byaruhanga and Kuteesa [24] also found that children with disabilities faced discrimination in the school setting and that there were insufficient funds to meet their academic needs. Education, discriminatory attitudes, and communication barriers for the deaf and blind were also found to impede education [22]. The results of the scoping study carried out by Talley and Brintell [25] indicated that children with disabilities may be overlooked as a priority in the education agenda because of a lack of staff and no budget allocation for inclusive education. They argued that little more than policy-making has been achieved overall.

### Article 8 (Awareness-raising)

Despite Article 8, because of high rates of non-awareness in the community, many articles cited that PWDs face extreme stigmatization and discrimination [10, 27]. This occurs not only on a community level, but also within families. For example, people who identify as albinos are oftentimes marginalized by their spouses and are believed to be cursed, leading to a greater rate of divorce. Additionally, they face adversity in gaining admission to primary school, and if they are accepted, they do not receive appropriate accommodations for their poor vision [28]. On a systematic level, many individuals are unaware of the needs of PWDs. For instance, only 50 per cent of healthcare workers were able to accurately define what palliative care constitutes for PWDs [29]. The Government has taken an initiative to observe numerous international awareness days in an effort to protect the rights of PWDs. Of great importance is the International Day of Persons with Disabilities, celebrated annually on December 3rd in Rwanda, in order to advance Articles 8 and 9 [30].

### Article 9 (Accessibility)

None of the policies and practices implemented under each of the UN CRPD Articles exist in isolation and are commonly intertwined with other Articles. The most commonly referenced example related to Article 9 (Accessibility), which is closely linked to awareness-raising, is a lack of accessibility due to stigmatization and discrimination. For example, Collins found that only four percent of buildings in Rwanda were accessible to PWDs [19]. Some buildings that stated they were accessible were not—the slope of the ramps put in place to access the buildings were incorrect and were hard for PWDs to traverse. According to Collins, there are many other programs being implemented in Rwanda, such as One-Stop Centre, Rwanda Bureau of Standards (RBS), and private sector developments, to address environmental accessibility needs [19]. Impassable roads and long commute times to reach health facilities were identified as concerns. Only 60 per cent of PWDs are in compliance with the Ministry of Health’s norm that states that all potential patients be located within a one-hour walking distance from a health center [31].

## Discussion

This is the first scoping study conducted that synthesizes evidence across a range of study designs to examine the realization of rights for PWDs in Rwanda. The findings of this study identify factors affecting the rights of PWDs in Rwanda. Overall, the scoping study identified a movement towards the realization of rights for PWDs in Rwanda since the country signed the UN CRPD. The publication dates of the identified studies show a movement towards the realization of rights for PWDs. There is a progression of publications, with a fewer number of articles published between 2008 to 2010, and more published as time progressed, with the most articles (over 50 per cent) published from 2014 to date.

Based on the reviewed evidence, publications highlighted efforts towards supporting the rights of PWDs, such as policy adoption and the establishment of organizations for PWDs. Research on the implementation of the UN CRPD was found to be in four main areas including Articles 7 (Children with Disabilities), 8 (Awareness-Raising), 9 (Accessibility), and 25 (Health). According to the Initial Report of Rwanda regarding the implementation of the CRPD, herein referred to as the Initial Report, the priorities of Rwanda in the implementation of the UN CRPD are Articles 9 (Accessibility), 24 (Education), 25 (Health), 28 (Adequate standard of living and social protection), and 29 (Participation in political and public life) [30]. All the priorities of Rwanda were included in the literature reviewed; however, Articles 24 and 28 were not among the top four most researched issues. The lack of integration of Article 28 into many of the identified studies, which provides essential economic context for discussions of disability in Rwanda, demonstrates that poverty may not be perceived as both a cause and consequence of disability or that social protection issues are not seen as integral to or intersecting with most every other sector. Adequate standard of living and social protection is a government priority and Rwanda has several government compensation programs that support the poor; however, there are a lack of programs specifically for PWDs [32]. More research needs to be done to determine the role income plays in moderating the impact of disability.

Article 25 (Health) was most discussed throughout the literature in part due to the country-wide health insurance, Mutuelle de Santé, as well as other significant government investments in health. Access to basic health services in Rwanda is nearly universal; 91 per cent of the population has access to health insurance coverage and health care is community-based [33]. Many other policies and programs exist within the Ministry of Health that address the rights of PWDs, such as the Non-Communicable Diseases Policy, a National Strategic Plan for the Prevention of Avoidable Blindness, and the Injury and Disability Unit. The amount of research focused on health for PWDs could also partially be explained by the fact that in Rwanda disability is typically viewed under the medical (instead of social) model. Under this model, several of the priorities fixed by Rwanda derive from this including the categorisation of PWDs, provision of appliances, and the focus on the physical adaptation of buildings.

Despite the many advances in policies and programming within the health sector and the embrace of evidence-based care by the Rwandan government, it is to imperative to note that the literature was all in agreement that mental health care in Rwanda remains an issue in need of more attention. Fear of stigmatization, poor community awareness of mental disorders, scarce resources in mental health care, underfinanced mental health services, and a lack of trained staff were all emphasized. A key factor in the need for greater investment in mental health services is that poverty and a history of trauma have dramatically shaped the mental health of many persons in Rwanda. The genocide continues to impact mental illness within the population. During the annual genocide memorial period (April-July), an increased number of traumatic episodes occur, indicating underlying mental health issues. Access to appropriate treatment remains difficult for those with mental health conditions because of widespread misunderstandings within the community about the nature of mental health conditions. Furthermore, only 11 per cent of the population lives in the capital Kigali where almost all mental health services are located. The Ministry of Health implemented the Mentoring and Enhanced Supervision at Health Centers for Mental Health program, a systematic approach to integrated mental healthcare that capacitates front-line public primary care health providers to care for people with mental disorders. However, this program does not exist in each district. Most primary care personnel have had little to no training in mental health.

Evidence highlighted the strengths of Rwandan communities in making advances in meeting the rights of children with disabilities, including being incredibly resourceful. This finding is congruent with published literature that traditional forms of social assistance, or community solidarity, known in Rwanda as “ubumwe”, are still strong features of everyday life. This community solidarity has previously been observed to hold great potential as a foundation for community-level responses to disability [22]. The Rwandan national strategy of emphasizing education is also an asset to inclusive education for children with disabilities. However, while the inclusion of children with disabilities is seen to be a current reality on paper—the Rwandan Constitution declares that, ‘The state has the duty to take special measures to facilitate the education of disabled people’–this is not seen in practice. Progressive national policies are not yielding results at the local level [22]. Only 10 per cent of children with disabilities are in school [34]. This finding resonates with the NCPD current Strategic Plan that indicates that “Rwanda has endorsed many legal instruments, 13 Ministerial Orders, sector policy & strategic plans are plentiful but implementation of several is still in early stages or unimplemented. At times a technical vision of ‘what to do’ is weak; at others funding is held up as the key constraint” [3].

This scoping study provides evidence that several Articles of the UN CRPD have yet to receive attention in research. For example, no evidence was found from the reviewed literature regarding a number of UN CRPD Articles, including Article 10 (Right to Life), Article 11 (Situations at risk and humanitarian emergency), Article 13 (Access to Justice), Article 14 (Liberty and security of the person), Article 15 (Freedom from Torture or Cruel, Inhuman or Degrading Treatment or Punishment), Article 16 (Freedom from Exploitation, Violence, and Abuse), Article 17 (Protecting the Integrity of the Person), Article 18 (Liberty of Movement and Nationality), Article 22 (Respect for Privacy), and Article 31 (Statistics and Data Collection). There are many plausible reasons why these particular Articles have not been mentioned in the literature. One possible explanation is that many of these Articles, such as Article 15, have legislation in place which addresses the right. The Constitution of the Republic of Rwanda, in its own Article 15, stipulates that “no person shall be subjected to torture, cruel or inhuman or degrading treatment; and no person may be subjected to experimentation without his/her informed consent”. With a lack of specific initiatives for PWDs in these areas, they may not be viewed as tangible issues for researchers to examine.

It can also be assumed that given the sensitive nature the of issues contained within many of the Articles, particularly right to life and violence, there may be barriers to carrying out research. Study approval and support may be more difficult to obtain since these discussions may be perceived as a vulnerable population as PWDs are commonly categorized. There are also more clear reasons for why Articles were not discussed, such as Article 31 (Statistics and Data Collection). Rwandan census data does not disaggregate disability statistics by age or impairment and the Initial UN CRPD report recognizes that much remains to be done in quantifying the needs of PWDs [30].

While many of these UN CRPD Articles were not recognized in the literature, perhaps not enough time has passed since the signing of the UN CRPD in 2008 for the successful implementation and integration of efforts in these sectors and for monitoring, evaluation and research to be carried out. These Articles could have been under-represented because of less activity in these sectors or fewer scientific publications stemming from the initiatives that exist. Another possibility is that there is a lack of researchers in Rwanda focusing on disability. Admittedly, there may be a more nuanced reason for this finding, such as a lack of investment in disability research from funders. More encouraging; however, is the finding that the fields of physical therapy and occupational therapy are growing—a school of occupational therapy opened in 2014. With an agenda for research, the scholars of these programs will surely contribute to the disability evidence base as the profession grows.

It is concerning that no research was found to exist concerning violence and abuse against PWDs in Rwanda or the institutionalization of PWDs. Given the existing stigmas, illuminated under the results presented under Article 8, PWDs are certainly being subjected to violence and abuse. One in five women in Rwanda have experienced sexual violence and women with no education are twice as likely to have experienced physical violence [17]. Women and children with disabilities are particularly vulnerable. While not addressed in the reviewed research, advancements in these areas are being made. The National Women’s Council is providing sensitization at a village level on the negative effects of violence against PWDs and Humanity and Inclusion (HI) is providing Mental Health and Sexual and Gender based violence programming. The NCPD, in conjunction with UNICEF, has also implemented an initiative to move children with disabilities living in institutions into family based care.

It is imperative to understand the underlying reasons why these Articles are not discussed in the literature. Once determined, it is foreseeable that an initiative to address all of the rights of PWDs will occur in an effort to ensure fair and equal treatment and portrayal throughout society. These findings also support the need for further research into the personal experiences of PWDs to identify human rights issues that they may be experiencing and what they view as priorities to be addressed.

### Limitations

The limitations of this scoping study include the inclusion of articles published only in English, which privileges the work of English authors over others, particularly those publishing in French, another official language of the country, and Kinyarwanda, the national language of Rwanda. As a scoping review aims to comprehensively synthesize evidence across a range of study designs in an area, a second limitation of this paper is that it provides a descriptive overview of the literature on the realization of rights for PWDs in Rwanda rather than a critical appraisal of the studies.

The limitations of using an approach guided by rights frameworks (i.e., UN CRPD) to position the study must also be taken into consideration. First, it must be recognized that the ratification of the UN CRPD alone will not bring about substantial or rapid change as local community and individual rights that are situated within the historical, political, and cultural contexts of the country must also be considered [35]. Therefore, in the case of Rwanda it must be considered how the rights identified in the UN CRPD may not resonate with PWDs living in Rwanda, as their culture and traditions influence how the rights and obligations are perceived.

As raised by seminal scholars in the disability field, it must be reiterated that this study was carried out by rehabilitation professionals located outside of Rwanda [36]. Thus, to translate the results into practice, PWDs and their organizations in Rwanda must be included. The first author is carrying out research in Rwanda that is participatory in nature with PWDs. The research will use these findings as a basis for setting a research agenda, building on the gaps in the literature identified and more importantly prioritized and chosen by PWDs living in Rwanda.

### Implications for future research

The findings of this study demonstrate there is a need for further research pertaining to the ways in which the rights of PWDs in Rwanda can be enhanced. As previously mentioned, there are significant gaps in the literature regarding human rights and justice matters for PWDs. The absence of literature regarding these aforementioned Articles points to the need for the disabilities and human rights sectors to further collaborate. Future research should also focus on advancing efforts currently being made to support the rights of PWDs in Rwanda. For example, progressive policies within the education system for children with disabilities should be further explored to ensure their carryover and adherence. This scoping study can serve as the platform on which future research and initiatives regarding the rights of PWDs in Rwanda can build.

## Conclusion

This scoping study explores the existing literature on the realization of rights for PWDs in Rwanda since the signing of the UN CRPD in 2008. Overall, Rwanda is committed to the realization of rights for PWDs—since the signing of the UN CRPD, key policies and programs have been enacted. The results of this study identified publications that detailed the steps that have been taken to realize the rights of PWDs in Rwanda and identified key factors affecting the acknowledgment of and action towards meeting the rights of PWDs in Rwanda, such as awareness, inclusive education standards, accessibility, and health services. Through the compilation and synthetization of this conclusive data, there is a demonstrated gap in the existing literature, and therefore a need for further advancement in research pertaining to the ways in which the rights of PWDs in Rwanda can be enhanced, particularly in the areas of justice, social protection, mental health, and violence and abuse. Collectively, this information can be used as a framework for future research on this topic, as well as to assist with the further implementation of supportive efforts for PWDs in Rwanda moving forward.

## Supporting information

S1 File Electronic search strategy for MEDLINE database. (DOCX)
