# Supplementary material for: Realization of the rights of persons with disabilities in Rwanda
Source: PLoS One. 2018 May 10;13(5):e0196347. doi: 10.1371/journal.pone.0196347 (PMC5944938; doi:10.1371/journal.pone.0196347)
Supplement: S1 File — (DOCX) [file pone.0196347.s001.docx]

**S1 File. Electronic search strategy for MEDLINE database.**

To identify as many relevant papers as possible for the research question: ‘What is known from the existing literature about the realization of the rights of PWDs since Rwanda signed the UN CRPD in 2008?’ numerous databases were searched. MEDLINE was one of the chosen databases because it is one of the most frequently used biomedical databases in addition to EMBASE. MEDLINE was also chosen because it is indexed and uses thesaurus terms to facilitate an easy and more complete search. To access MEDLINE, the search interface PubMed was chosen because it is very widely used and is freely available.

The key constructs in the research question were used to develop a search string, which included an extensive collection of search terms. This study used both standardized subject, which are called Medical Subject Headings (MeSH terms) in MEDLINE, and free-text terms. The free-text terms in addition to MeSH terms were used because not all papers that are searched through PubMed have already been indexed. To economize the search string, truncation [adding * to a word stem] was used in combination with free-text words.

The relevant MeSH terms, included: disabled children, disabled persons, Rwanda, civil rights, rehabilitation, health services accessibility, architectural accessibility.

The free-text terms included: Rwanda, disab*, impair*, handicap*, rehab*, access*, rights, persons living with disab*, person* with disab*, people with disab*, disab* person.

Using the above MeSH and free-text terms [tw], the following search was performed:

1. disabled children [MeSH]
2. disabled persons [MeSH]
3. Rwanda [MeSH]
4. civil rights [MeSH]
5. rehabilitation [MeSH]
6. health services accessibility [MeSH]
7. architectural accessibility [MeSH]
8. rights [tw]
9. Rwanda [tw]
10. rehab*[tw]
11. access* [tw]
12. disab* OR impair* OR handicap* OR persons living with disab* OR person* with disab* OR people with disab* OR disab* person [tw]
13. #1 OR #2 OR #12
14. #3 OR #9
15. #4 OR #8
16. #5 OR #10
17. #6 OR #7 OR #11
18. #13 AND #14
19. #18 AND #15
20. #18 AND #16
21. #18 AND #17

Limits used for inclusion:

(a) Published during the years 2008 to 2017. The year 2008 was chosen because that is when Rwanda signed the UN CRPD.

(b) Written in the English language.

(c) Peer reviewed.

(d) Publication is about disability or contains information on at least one of the disability categories (physically disabled, sight-impaired, deaf-and-dumb, mentally disabled) outlined by the Rwandan census.
